# Bushen Jianpi Formula Combined with Entecavir for the Treatment of HBeAg-Negative Chronic Hepatitis B: A Multicenter, Randomized, Double-Blind, Placebo-Controlled Trial

**DOI:** 10.1155/2022/6097221

**Published:** 2022-03-25

**Authors:** Jing-Hao Zhang, Xin Zhang, Zhen-Hua Zhou, Xiao-Jun Zhu, Chao Zheng, Man Li, Shu-Gen Jin, De-Wen Mao, Jing-Dong Xue, Wei-Bing Shi, Xiao-Ling Chi, Xian-Bo Wang, Xiao-Dong Li, Yong Li, Hui Wang, Qin Li, Da-Qiao Zhou, Cheng-Bao Wang, Chang-He Shi, Cheng-Zhong Li, Jian-Hua Wu, Xiao-Ni Kong, Xue-Hua Sun, Yue-Qiu Gao

**Affiliations:** ^1^Department of Hepatopathy, Shuguang Hospital, Affiliated to Shanghai University of Traditional Chinese Medicine, Shanghai 201203, China; ^2^Laboratory of Cellular Immunity, Shanghai Key Laboratory of Traditional Chinese Medicine, Affiliated to Shanghai University of Traditional Chinese Medicine, Shanghai 201203, China; ^3^Department of Hepatology, The First Affiliated Hospital of Guangxi University of Chinese Medicine, Nanning 530023, China; ^4^Department of Hepatology, Shaanxi Hospital of Traditional Chinese Medicine, Xi'an 710003, China; ^5^Department of Infectious Diseases, First Affiliated Hospital of Anhui University of Traditional Chinese Medicine, Hefei 230031, China; ^6^Department of Hepatology, Guangdong Hospital of Traditional Chinese Medicine, Guangzhou 510006, China; ^7^Department of Integrated TCM and Western Medicine, Ditan Hospital Affiliated of Capital Medical University, Beijing 100015, China; ^8^Department of Hepatology, Hubei Provincial Hospital of Traditional Chinese Medicine, Wuhan 430061, China; ^9^Department of Hepatology, The Affiliated Hospital of Shandong University of Traditional Chinese Medicine, Jinan 250011, China; ^10^Department of Infectious Diseases, Ruijin Hospital, Shanghai Jiao Tong University School of Medicine, Shanghai 200025, China; ^11^Department of Hepatology, Department of Infectious Disease, Fuzhou Infectious Diseases Hospital, Fuzhou 350000, China; ^12^Department of Hepatology, Shenzhen Traditional Chinese Medicine Hospital, Shenzhen 518033, China; ^13^Department of Infectious Diseases, Linyi People's Hospital, Linyi 276003, China; ^14^Qingdao Liver Diseases Institute, Qingdao Hospital of Infectious Diseases, Qingdao 266033, China; ^15^Department of Infectious Diseases, The First Affiliated Hospital of Naval Medical University, Shanghai 200433, China; ^16^Department of Hepatopathy, Xiamen Hospital of Traditional Chinese Medicine, Xiamen 361001, China; ^17^Central Laboratory, Shuguang Hospital, Affiliated to Shanghai University of Traditional Chinese Medicine, Shanghai 201203, China

## Abstract

**Background:**

Bushen Jianpi formula (BSJPF, also known as Lingmao formula) is a traditional Chinese medicine for chronic hepatitis B (CHB). The previous study has suggested that the treatment combination of BSJPF and entecavir (ETV) can achieve a significant loss of hepatitis B e antigen (HBeAg) and a significant decrease in serum level of hepatitis B virus (HBV) DNA in HBeAg-positive CHB patients with mildly elevated alanine aminotransferase.

**Objective:**

This study aimed to evaluate the efficacy and safety of BSJPF combined with ETV for treating HBeAg-negative CHB patients.

**Methods:**

A total of 640 patients were assigned randomly to the treatment group (receiving BSJPF combined with ETV for 96 weeks) or the control group (receiving a placebo combined with ETV for 96 weeks) in a 1 : 1 ratio. The primary endpoints are the rate of loss of hepatitis B surface antigen (HBsAg). The secondary outcomes included the rate of decrease in the HBsAg concentration to ≥1 lg·IU/mL, the HBV DNA suppression, the decline of the level of covalently closed circular DNA (cccDNA) in the liver, histological improvements, and the rate of ALT normalization.

**Results:**

The rate of HBsAg loss in the treatment group was significantly higher than that of the control group (5.5% *versus* 1.8%, *P*=0.031). There were 11.1% of patients in the treatment group who recorded a reduction in HBsAg ≥1 lg·IU/mL, which is better than 5.9% of patients in the control group (*P*=0.043). There was no significant difference between the two groups with regard to the rate of HBV DNA clearance, the reduction in intrahepatic cccDNA, and the rate of ALT normalization (*P* > 0.05). The rate of liver fibrosis improvement in the treatment group was better than that of the control group (35.5% *versus* 11.8%, *P*=0.031), but there was no difference in necroinflammatory improvement (*P* > 0.05). The adverse events (AEs) were similar between the two groups, except for the abnormal kidney function, with 2.2% in the control group and 0.0% in the treatment group (*P*=0.028).

**Conclusion:**

The combination of BSJPF and ETV can increase the rate of HBsAg loss and the rate of histological fibrosis improvement without serious adverse events in CHB patients. *Trial Registration*. This trial is registered with ChiCTR-IOR-16009880 on November 16, 2016—retrospectively registered, http://www.chictr.org.cn/showproj.aspx?proj=16836.

## 1. Backgrounds

Hepatitis B virus (HBV) infection is a public health problem worldwide, with approximately 257 million people suffering from chronic HBV infection and causing 887,000 deaths around the world every year [1, 2]. Although the prevalence of being positive in hepatitis B surface antigen (HBsAg) among the patients that are under the age of 30 has declined from 10.1% to 2.6% in China [3], it is estimated that there are still about 70 million HBsAg carriers and 20–30 million chronic hepatitis B (CHB) patients at present [4].

Currently, the treatment goals for CHB are to reduce the decompensation of liver cirrhosis, liver dysfunction, and hepatocellular carcinoma (HCC), to improve the quality of life of patients, and to prolong their survival time [4–6].

HBsAg clearance is closely associated with improved liver pathology and survival time [7], and thus, it is the ideal therapeutic goal. Numerous researches have shown that the serum HBsAg quantification could be used to assess the degree of HBV replication activity in the liver, evaluate the progression of CHB [8, 9], predict the patient's response during treatment and after drug withdrawal [10, 11], discover drug-resistant mutant strains, and judge virological breakthroughs [12]. The earlier the clearance of HBsAg is achieved, the better the long-term prognosis of patients with chronic hepatitis B. About 80% of CHB patients receive treatment with nucleoside analogues (NAs), and long-term use of NAs can decrease covalently closed circular DNA (cccDNA) and HBsAg levels [13]. However, with this approach, the HBsAg clearance rate is only 0–3% [4] and the risk of virological relapse is relatively high [14, 15]. Therefore, new treatment strategies are urgently needed to improve the rate of HBsAg clearance and to achieve a clinical cure for CHB.

Traditional Chinese medicine (TCM) has been used in the treatment of CHB for hundreds of decades in China and other parts of the world. Research has shown that some Chinese herbal formulae might be able to inhibit HBV DNA replication, improve the rate of hepatitis B e antigen (HBeAg) clearance, and improve liver function. Such formulae have included Xiao Chai Hu Tang, Xiao Yao San, and Long Dan Xie Gan Tang [16]. The previous study has suggested that the combination of Bushen Jianpi formula (BSJPF, also known as Lingmao formula) and entecavir (ETV) could result in significant loss of HBeAg and a significant decrease in the serum level of HBV DNA in HBeAg-positive CHB patients with mildly elevated alanine aminotransferase [17]. However, no additional evidence-based data regarding the efficacy and safety of BSJPF for HBeAg-negative CHB patients are currently available. To address this, we conducted this multicenter, randomized, double-blind, placebo-controlled clinical trial to determine the effects of BSJPF in HBeAg-negative CHB patients.

## 2. Methods

### 2.1. Study Design

This study was a multicenter, randomized, double-blind, placebo-controlled clinical trial. All patients enrolled were treated with 0.5 mg ETV daily and were randomly assigned to receive BSJPF or a placebo (15 mg orally) twice a day for 96 weeks. ETV was purchased from CTTQ Pharmaceutical, Jiangsu Province, China (Drug Manufacturing Certificate ID: H20100019). BSJPF was composed of Yin Yang Huo (*Epimedium brevicornum* Maxim. leaf), Mao Zhua Cao (*Ranunculus ternatus* Thunb. root), Huang Qi (*Astragalus membranaceus* [Fisch.] Bge. *var. Mongholicus* [Bge.] Hsiao. root), Bai zhu (*Atractylodes macrocephala* Koidz. rhizome), Sheng Ma (*Cimicifugae rhizoma*), Ku Shen (*Sophora flavescens* Ait. root), Qing Pi (*Citrus reticulata* Blanco. immature fruit peel), Dan Pi (*Paeonia suffruticosa* Andr. peel), Lian Qiao (*Forsythia suspensa* [Thunb.] Vahl. fruit), and Xian He Cao (*Agrimonia pilosa* Ledeb.). The concentrated granules of the herbs described above were provided by Shenzhen 999 Pharmaceutical Co. Ltd. The source, preparation, and quality control of granules are strictly in accordance with the relevant standards of Chinese Pharmacopoeia. The placebo was also in a concentrated granule form and was composed of 10% of each herb in BSJPF, a bittering agent, and a pharmaceutical excipient provided by the same company. The bittering agent and pharmaceutical excipient were used following the regulations on the management of pharmaceutical excipients and hygiene standards for use in food additives in China. The placebo shared the same package, label, appearance, and taste as BSJPF.

### 2.2. Randomization and Blinding

Randomization was centralized through the Central Randomization System for Clinical Research (Web Edition, http://www.tcmcec.net/crivrs/) developed by the Clinical Evaluation Center of the China Academy of Chinese Medical Science. After baseline assessment and signing the informed consent, 640 patients were randomly assigned to the treatment group or the control group in a 1 : 1 ratio, with a unique random number. Each random number in the central randomization system matched a number for the Chinese herb medicine. Physicians and patients were blinded to the type of medicine patients received.

### 2.3. Data Collection and Management

All the raw data of patients were collected through a printed case report form and were logged twice by two different data managers in the clinical research data management system (Web Edition, http://www.tcmcec.net/wcr/) developed by the Clinical Evaluation Center of the China Academy of Chinese Medical Science.

### 2.4. Ethical Approval

This study was conducted following the ethical principles of the Declaration of Helsinki. The study protocol was approved by the Ethics Committee of Shuguang Hospital, Shanghai University of Traditional Chinese Medicine (No. 2012-220-36-01; Shanghai, China). Written informed consent was obtained from all enrolled patients. The trial was registered in the Chinese Clinical Trial Registry on November 16, 2016, and the clinical trial number is registered as ChiCTR-IOR-16009880.

### 2.5. Patient Eligibility

All the patients were screened between June 20, 2013, and July 12, 2016. The eligibility criteria included the following: (1) patients with a history of HBV or those who were HBsAg-positive for more than 6 months; (2) patients with positivity for HBsAg or HBV DNA; (3) patients with persistent HBeAg negativity or anti-HBe positivity; (4) patients with an HBV DNA concentration of 1 × 10^4^ copies/mL or higher by polymerase chain reaction (PCR) assay at least 4 weeks before screening [18]; (5) patients with an ALT concentration two times of the upper limit of the normal range (2 ULN) or greater, or 1 ULN ≤ ALT < 2 ULN and Knodell histology activity index of 4 points or greater at least 4 weeks before screening; (6) patients who had not received any treatment with any NAs or interferon at least 12 months before screening; and (7) patients aged between 18 and 65 years.

The exclusion criteria included the following: (1) patients coinfected with other hepatitis viruses; (2) patients with HCC and other forms of liver disease; (3) patients with other severe primary disease or mental disorder disease; (4) patients with an allergic constitution or multiple drug allergy; and (5) patients who were pregnant or lactating.

### 2.6. Follow-Up

Patients enrolled were asked to visit 12, 24, 36, 48, 60, 72, 84, and 96 weeks after randomization. The parameters measured were HBV DNA, HBV serology, liver function, alpha-fetoprotein (AFP), ultrasonography, and liver stiffness (kPa score). Histopathological evaluations and an assessment of safety were carried out after randomization and at 96 weeks.

### 2.7. Efficacy Endpoints

The primary endpoints are the rate of HBsAg loss. The secondary outcomes included the rate of decrease in the HBsAg concentration to ≥1 lg·IU/mL, the HBV DNA suppression, the decrease in cccDNA in the liver, the histological response (defined as an improvement of at least one grade in the Scheuer necroinflammatory grade or in the fibrosis stage), and the biochemical response (the rate of ALT normalization). 20% of patients were demanded to undergo liver biopsy for histopathological evaluation. The liver biopsy sample was required a 1.5–2.5 cm length and at least 6 portal tracts.

All the blood and liver tissue samples were delivered to a third-party testing agency (Shanghai Adicon Clinical Laboratories, Inc., College of American Pathologists-Certified) for centralized detection. The serum HBsAg was measured by the Elecsys HBsAg II quant assay (Roche Diagnostics), and HBV DNA and intrahepatic cccDNA were detected by rolling cycle amplification polymerase chain reaction with QIAamp DNA Mini Kit and QIAamp DNA FFPE Tissue Kit (Qiagen).

### 2.8. Assessment of Safety

All the patients were questioned about any changes in health during the follow-up. For each patient, an electrocardiogram (ECG), complete blood count (CBC), stool analysis, urine test, and kidney function test (for serum creatinine and urea nitrogen) were performed after the enrollment and at 96 weeks. Adverse events (AEs) were documented by the patients' physicians at each follow-up point.

### 2.9. Statistical Analyses

The sample size was calculated based on a 2% HBsAg-negative conversion rate of HBeAg-negative patients treated with ETV [19] and an expected 6% HBsAg-negative conversion rate for patients treated with ETV and BSJPF. An alpha error of 0.05 and a study power of 80% (beta = 0.20) were considered, suggesting that 256 patients were required in each group. Assuming a dropout rate of 20%, it was calculated that a total of 640 patients, with 320 patients in each group, were required. The rate of HBsAg-negative conversion, the rate of decrease in the HBsAg concentration ≥1 lg·IU/mL, and the rate of undetectable HBV DNA between the two groups were assessed by the chi-square test. Declining cccDNA levels were assessed by the paired *t*-test. The Mann–Whitney *U*-test was used to compare the histological improvements between the groups. For the assessment of safety, a comparison of the differences between the groups was conducted by Pearson's chi-square test or Fisher's exact test. Logistic regression was used to analyze the factors affecting the decline of HBsAg. Statistical analyses were performed using the SPSS v24.0 (IBM; Armonk, NY, USA). All *p* values were two-sided with a significance level of 0.05.

## 3. Results

### 3.1. Demographics

**Figure 1:**
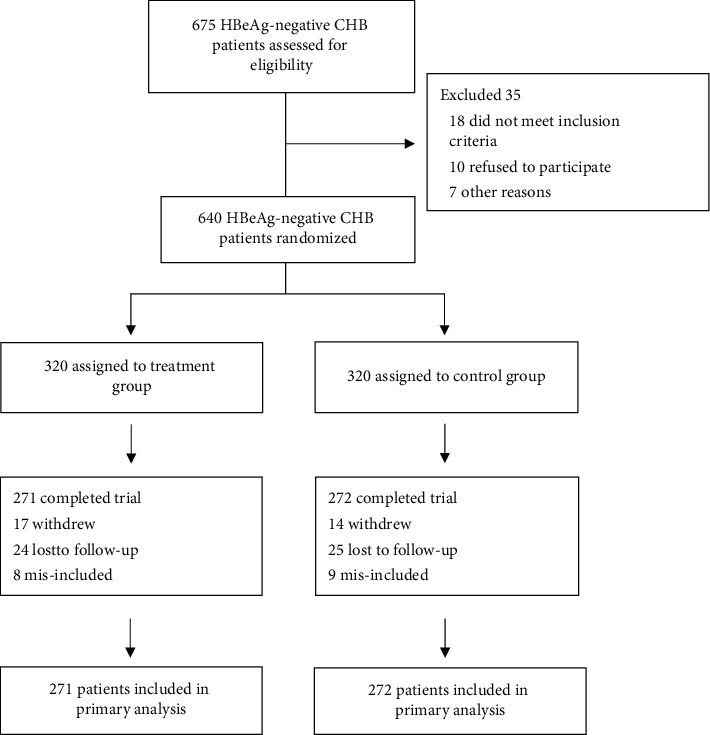
Flow diagram of the randomized clinical trial.

Patients were recruited from 15 hospitals in China between June 20, 2013, and July 12, 2016. Patients (*n* = 640) were randomly assigned to the treatment group or the control group (320 patients in each group) in a double-blinded manner. In total, 271 patients in the treatment group and 272 patients in the control group completed the trial and were included for analysis. Among them, a total of 179 patients had liver biopsy at baseline, and 31 patients in the treatment group and 34 patients in the control group underwent liver biopsy for histopathological evaluation at the beginning as the baseline and at the end of the trial. The baseline characteristics of the patients in the two groups are shown in Table 1, and no significant difference was observed between the two groups. A flow diagram of the trial is shown in Figure 1.

### 3.2. HBsAg Clearance

**Figure 2:**
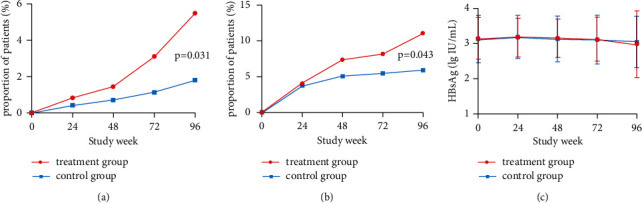
HBsAg clearance by visit week. (a)Proportion of patients achieving HBsAg loss by study visit. (b) Proportion of patients recorded a reduction in HBsAg ≥ 1 lg IU/mL by study visit. (c) The serum HBsAg level in two groups by study visit.

As shown in Table 2, the rate of HBsAg loss in the treatment group was 5.5%, which is higher than the rate of 1.8% in the control group (*P*=0.031; Figure 2(a)). The percentage of patients who recorded a reduction in the serum level of HBsAg that is more than 1 log10 copies/mL was 11.1% in the treatment group and 5.9% in the control group (*P*=0.043; Figure 2(b)). It had no significant difference in the serum HBsAg level between the two groups by study visit (*P* > 0.05; Figure 2(c)).

**Figure 3:**
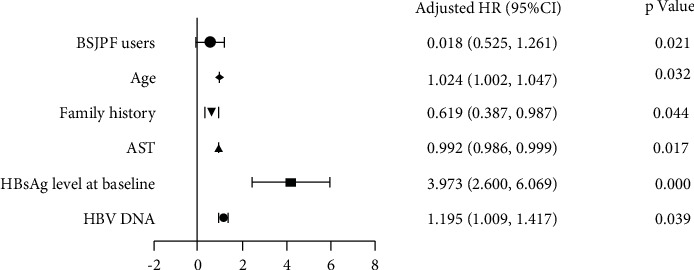
Multivariate analysis on the factors that may affect the decline of HBsAg.

Univariate analysis was performed on the factors that may affect the decline of HBsAg. The results showed that gender (*R* = −0.091, *P*=0.048), baseline HBsAg level (*R* = 0.502, *P* < 0.001), HBV DNA level (*R* = 0.163, *P* < 0.001), HBV genotype (*R* = −0.115, *P*=0.013), and family history (*R* = −0.097, *P*=0.037) had a linear correlation with the decreased level of HBsAg after treatment. The results of multivariate analysis showed that baseline HBsAg level (lg value), age, family history, AST, DNA level, and group were the influencing factors of HBsAg decline (*P* < 0.05; Figure 3).

### 3.3. Viral Suppression

**Figure 4:**
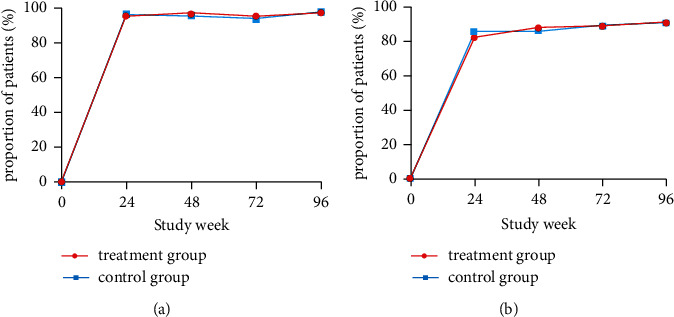
HBV DNA suppression and alanine aminotransferase (ALT) normalization by visit week. (a) Proportion of patients with undetectable levels of HBV DNA by study visit. (b) Proportion of patients achieving ALT normalization by study visit.

**Figure 5:**
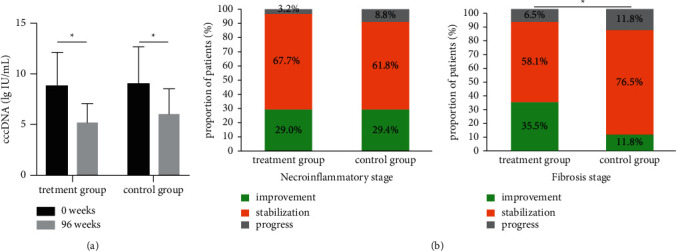
Decrease in intrahepatic cccDNA and histological Response. (a) The cccDNA in the liver at the baseline and at the end of the trial. (b) The Scheuer necroinflammatory grade and the fibrosis stage at the baseline and at the end of the trial.  ^*∗*^*p* < 0.05.

The difference between the two groups with regard to the virological response is shown in Table 2. Undetectable levels of HBV DNA were identified in 97.8% of patients in the treatment group and 98.2% of patients in the control group (*P*=0.770; Figure 4(a)). The mean decrease in cccDNA in the liver from baseline was not significantly different between the treatment group and the control group (3.66 *versus* 3.06 lg IU/mL, *P*=0.636; Figure 5(a)).

### 3.4. Histological Response

A total of 65 patients had liver biopsy specimens assessed both at the beginning of the trial as the baseline and after 96 weeks (31 in the treatment group and 34 in the control group). Histopathological evaluation of the liver samples showed that 29.0% of patients in the treatment group and 29.4% of patients in the control group achieved necroinflammatory improvement (*P*=0.766). Furthermore, 35.5% of patients in the treatment group and 11.8% of patients in the control group achieved an improvement in fibrosis (*P*=0.031; Table 2, Figure 5(b)).

### 3.5. Biochemical Response

A total of 437 patients showed ALT > 1 ULN at baseline (219 patients in the treatment group and 218 patients in the control group). The percentage of ALT normalization after 96 weeks was 91.8% in the treatment group *versus* 91.3% in the control group (*P*=0.868; Table 2). No significant difference was shown in the percentage of ALT normalization between the two groups by different study visits (*P* > 0.05; Figure 4(b)).

### 3.6. Safety

The frequency of AEs during the follow-up period between the two groups was not significantly different (Table 3). All the AEs were mild and tolerable. The most frequent AEs were abdominal pain, diarrhea, gingival bleeding, fatigue, and fever. Abnormal results for the CBC, stool analysis, urine test, and ECG were reported in both groups, and there were no significant differences between the groups. However, there was a significant difference with regard to kidney function as no patients in the treatment group had abnormal kidney function during the follow-up period compared with six patients in the control group (*P*=0.028).

## 4. Discussion

The treatment guidelines worldwide for CHB have highlighted the importance of antiviral therapy and recommend NAs and pegylated interferon (PEG-IFN) as the first-line treatments. NAs can effectively inhibit HBV replication; long-term antiviral therapy can reverse liver fibrosis and cirrhosis and reduce the incidence of primary liver cancer [20–22]. However, to date, it is impossible to achieve complete HBsAg and cccDNA clearance using these therapies along. In addition, problems, such as drug resistance, relapse after drug withdrawal, and poor patient compliance, have been reported. PEG-IFN combined with NAs can result in a functional cure for some patients and improve the negative conversion rate of HBsAg [23–26]; however, the adverse reactions, such as flu-like symptoms, bone marrow suppression, and mental health abnormalities, have limited the clinical application of PEG-IFN.

Many studies have shown that traditional Chinese herbs have anti-HBV effects. For example, the total ethanol extract and saponins from *Radix Astragali* have been shown to suppress the secretion of HBsAg and HBeAg in the HepG2.2.15 cell line [27]. In addition, matrine extracted from *Sophorae flavescentis* has been shown to have anti-HBV activity and to improve liver function by regulating the Toll-like receptor 9 signaling pathway [28–30]. Furthermore, an extract of *Paeonia suffruticosa* Andr. has been shown to suppress the secretion of HBV antigens and to reduce HBV DNA level in the serum and livers of ducklings challenged with duck hepatitis B virus [31].

Here, our study demonstrated that BSJPF combined with ETV could increase the rate of HBsAg loss and the rate of serum HBsAg decreased to ≥1 lg·IU/mL. Our previous studies have shown that BSJPF had positive effects on CHB patients by reducing serum ALT and HBV DNA levels through decreasing the percentage of CD4+CD25+T cells and increasing the expression level of IFN-*γ* in CD4+ T cells [32]. Furthermore, it could also promote the reduction in HBsAg level and the clearance of HBeAg in CHB patients by regulating the differentiation of B-cell subsets through increasing the B-cell-activating factor, the frequency of Bm1, and CD24+CD27-swiched B cells [33], and by increasing the Th1 and DC frequencies and decreasing Treg frequency and downregulating PD-L1 levels on DCs [34]. It was shown in the *in vitro* study that BSJPF could decrease the microtubule-associated protein 1 light-chain 3 beta II/I and inhibit starvation-induced autophagy in HepG 2.2.15 cells [35].

Liver fibrosis is a common complication of CHB. It is the intermediate stage of the CHB progression towards liver cirrhosis and liver cancer. Currently, there are no effective biological or chemical drugs that can be used to treat liver fibrosis. TCM is considered comprehensive treatment since it can produce the effects through multichannel, multi-target, and multilevel routes [36]. TCM for CHB has been shown to block and reverse hepatic fibrosis and to slow the progression of hepatitis B-related liver diseases [37]. The histopathological evaluation conducted in this study showed that BSJPF combined with ETV could improve liver fibrosis in CHB patients. Our previous studies showed that the anti-fibrosis mechanism of TCM formula lay in reducing the collagen expression of types I, III, and IV in hepatocytes and those of fibrotic hepatic stellate cells [38], inhibiting the expression of tissue inhibitor of metalloproteinases 1 gene expression in HSC, promoting the degradation of collagen [39], and reducing the expression of osteopontin induced by TGF-*β*1 through inhibiting the PI3K/PKB signaling pathway [40].

Recently, a study assessed drug-induced liver injury in mainland China and showed that the leading class of implicated drugs was TCMs, as well as herbal and dietary supplements [41], which focused the attention on the safety of TCMs once again. In our study, there was no significant difference between the two groups with regard to AEs, except for abnormal kidney function, which was found to be higher in the control group. Whether BSJPF may have a protecting effect on kidney function needs to be clarified in future research.

Our study had some limitations. The follow-up period was only 96 weeks, and the sample size was relatively small. A longer-term study with an extended sample size is needed to further confirm the clinical efficacy of BSJPF. In addition, BSJPF consists of ten Chinese herbs, resulting in a complex extract with many active compounds. Thus, the anti-HBV mechanism of BSJPF is not clear and requires further analysis. In addition, although it is recommended that HBeAg-negative CHB patients treated with ETV can stop NA treatment when HBsAg disappears and HBV DNA is undetectable [42], the sustained anti-HBV response is still limited [43]. Therefore, the effect of BSJPF after stopping NA treatment should be studied further.

## 5. Conclusions

Taken together, this nationwide multicenter study, involving 15 centers, has provided evidence for the effectiveness of BSJPF to increase the HBsAg-negative conversion rate and the rate of histological improvement in CHB patients, suggesting that it could be an alternative therapy for HBeAg-negative CHB.

## Figures and Tables

**Table 1:** Demographics and baseline characteristics of the patients.

Characteristic	Treatment group (*n* = 271)	Control group (*n* = 272)	*P*
Age (years)^†^	42.36 ± 11.23	39.61 ± 12.40	0.631
Male, *n* (%)	188 (69.89)	162 (62.79)	0.31
Duration of illness (years)^†^	11.17 ± 6.22	10.7 ± 6.28	0.43
MTCT, *n* (%)	63 (23.42)	49 (18.99)	0.52
Family history, *n* (%)	99 (36.8)	82 (31.78)	0.29
Smoking, *n* (%)	25 (9.29)	26 (10.08)	0.78
Alcohol consumption, *n* (%)	38 (14.13)	28 (10.85)	0.534
BMI^†^	24.08 ± 14.98	24.06 ± 13.04	0.99
ALT (U/L)^†^	73.31 ± 15.42	72.65 ± 16.29	0.68
AST (U/L)^†^	49.51 ± 19.21	51.93 ± 20.08	0.31
HBsAg (lg IU/mL)^†^	3.15 ± 0.59	3.12 ± 0.67	0.87
HBV DNA (lg IU/mL)^†^	6.90 ± 1.31	6.84 ± 1.45	0.82
cccDNA (lg IU/mL)^†^	8.89 ± 3.22	9.10 ± 3.35	0.77
HBV genotypes			0.312
B	113 (42.8)	139 (48.4)	
C	152 (56.5)	144 (50.2)	
D	0 (0)	1 (0.3)	
G	2 (0.7)	3 (1.0)	
kPa score^†^	9.99 ± 6.97	10.16 ± 8.16	0.719
Grade of necroinflammation			0.733
G1	5 (5.7)	7 (7.7)	
G1–2	0 (0)	1 (1.1)	
G2	71 (80.7)	72 (79.1)	
G2–3	2 (2.3)	1 (1.1)	
G3	10 (11.4)	10 (11.0)	
Stage of fibrosis			0.183
S0	1 (1.1)	0 (0.0)	
S1	16 (18.2)	21 (23.3)	
S1–2	1 (1.1)	2 (2.2)	
S2	49 (55.7)	54 (60.0)	
S2–3	1 (1.1)	1 (1.1)	
S3	20 (22.7)	9 (10.0)	
S4	0 (0.0)	3 (3.3)	

Values in parentheses are percentages; ^†^value is the mean ± standard deviation. ALT, alanine aminotransferase; AST, aspartate aminotransferase; BMI, body mass index; MTCT, mother-to-child transfection.

**Table 2:** Comparison of efficacy endpoints between the treatment and control groups.

Endpoint	Treatment group (*n* = 271)	Control group (*n* = 272)	*P*
HBsAg loss	15 (5.5)	5 (1.8)	0.031
Decrease in HBsAg to ≥1 lg·IU/mL	30 (11.1)	16 (5.9)	0.043
HBV DNA suppression	261 (97.8)	267 (98.2)	0.770
Mean decrease in cccDNA (lg IU/mL)^†‡^	3.66 ± 1.75	3.06 ± 1.32	0.636
Necroinflammatory improvement^‡^	9 (29.0)	10 (29.4)	0.766
Fibrosis improvement^‡^	11 (35.5)	4 (11.8)	0.031
ALT normalization^§^	201 (91.8)	199 (91.3)	0.868

Values in parentheses are percentages; ^†^value is the mean ± standard deviation. ^‡^A total of 65 patients had liver biopsy specimens assessed both at baseline and at 96 weeks (31 in the treatment group and 34 in the control group). ^§^A total of 437 patients had abnormal ALT levels at baseline (219 patients in the treatment group and 218 patients in the control group).

**Table 3:** Adverse events reported during the follow-up period.

Adverse event	Treatment group (*n* = 271)	Control group (*n* = 272)	*P*
Abnormal CBC	51 (18.8)	37 (13.6)	0.111
Abnormal kidney function	0 (0.0)	6 (2.2)	0.028
Abnormal stool analysis	7 (2.6)	2 (0.7)	0.156
Abnormal urine test	47 (17.3)	31 (11.4)	0.069
Abnormal ECG	3 (1.1)	4 (1.5)	0.643
Diarrhea	19 (7.0)	23 (8.5)	0.630
Gingival bleeding	22 (8.1)	32 (11.8)	0.197
Fatigue	28 (10.3)	26 (9.6)	0.776
Fever	6 (2.2)	8 (3.7)	0.448
Abdominal pain	42 (15.5)	59 (21.7)	0.077
Any other complaints	3 (1.2)	8 (3.0)	0.216

CBC, complete blood count; ECG, electrocardiogram. Other complaints included injury, extrauterine pregnancy, and intracranial tumor.

## Data Availability

The data used to support the findings of this study are available from the corresponding author upon request.

## Abbreviations

ALT: Alanine aminotransferase
AFP: Alpha-fetoprotein
AEs: Adverse events
BSJPF: Bushen Jianpi formula
CBC: Complete blood count
CHB: Chronic hepatitis B
cccDNA: Covalently closed circular DNA
ECG: Electrocardiogram
ETV: Entecavir
HBV: Hepatitis B virus
HBeAg: Hepatitis B e antigen
HBsAg: Hepatitis B surface antigen
HCC: Hepatocellular carcinoma
HSC: Hepatic stellate cells
NAs: Nucleos(t)ide analogues
PEG-IFN: Poly pegylated interferon
TCM: Traditional Chinese medicine
ULN: Upper limit of normal.
